# Early Pregnancy Exposure to Rare Earth Elements and Risk of Gestational Diabetes Mellitus: A Nested Case-Control Study

**DOI:** 10.3389/fendo.2021.774142

**Published:** 2021-12-20

**Authors:** Xiangrong Xu, Yuanyuan Wang, Na Han, Xiangming Yang, Yuelong Ji, Jue Liu, Chuyao Jin, Lizi Lin, Shuang Zhou, Shusheng Luo, Heling Bao, Zheng Liu, Bin Wang, Lailai Yan, Hai-Jun Wang, Xu Ma

**Affiliations:** ^1^ Department of Maternal and Child Health, School of Public Health, Peking University, Beijing, China; ^2^ Environmental and Spatial Epidemiology Research Center, National Human Genetic Resources Center, Beijing, China; ^3^ Human Genetic Resources Center, National Research Institute for Family Planning, Beijing, China; ^4^ Obstetrical Department, Tongzhou Maternal and Child Health Hospital of Beijing, Beijing, China; ^5^ Department of Epidemiology and Biostatistics, School of Public Health, Peking University, Beijing, China; ^6^ Institute of Reproductive and Child Health, Peking University/Key Laboratory of Reproductive Health, National Health and Family Planning Commission of the People’s Republic of China, Beijing, China; ^7^ Department of Laboratorial Science and Technology, School of Public Health, Peking University, Beijing, China

**Keywords:** rare earth elements (REEs), gestational diabetes mellitus (GDM), serum, early pregnancy, weighted quantile sum (WQS)

## Abstract

**Objective:**

The extensive use of rare earth elements (REEs) in many technologies was found to have effects on human health, but the association between early pregnancy exposure to REEs and gestational diabetes mellitus (GDM) is still unknown.

**Methods:**

This nested case-control study involved 200 pregnant women with GDM and 200 healthy pregnant women from the Peking University Birth Cohort in Tongzhou. We examined the serum concentrations of 14 REEs during early pregnancy and analyzed their associations with the risk of GDM.

**Results:**

When the elements were considered individually in the logistic regression model, no significant associations were found between REEs and GDM, after adjusting for confounding variables (*P* > 0.05). In weighted quantile sum (WQS) regression, each quartile decrease in the mixture index for REEs resulted in a 1.67-fold (95% CI: 1.12-2.49) increased risk of GDM. Neodymium (Nd), Praseodymium (Pr), and Lanthanum (La) were the most important contributors in the mixture.

**Conclusion:**

The study findings indicated that early pregnancy exposure to lower levels of REE mixture was associated with an increased risk of GDM, and Nd, Pr, and La exhibited the strongest effects in the mixture.

## Introduction

Gestational diabetes mellitus (GDM), defined as any degree of glucose intolerance with onset or first recognition during pregnancy (1), affects a considerable number of pregnant women worldwide (2). GDM is associated with many short- and long-term adverse health outcomes for mothers and their offspring. Mothers with GDM are more likely to develop preeclampsia during pregnancy and type 2 diabetes mellitus (T2DM) 5-10 years after delivery (3, 4). The offspring of mothers with GDM have a higher incidence of macrosomia and a higher risk of diabetes and other cardiometabolic diseases later in life (5–7). Both genetic susceptibility and lifestyle risk factors have been shown to play a role in the etiology of GDM, yet the impact of environmental exposure remains unclear (8–10). Recently, the effects of environmental metal exposure on GDM risk have attracted increasing attention worldwide. Different metals exhibited protective (11–13) or deleterious (14–17) effects on the development of GDM.

Rare earth elements (REEs) have gained worldwide attention due to the rapid development of industrial, agricultural and medical technologies in the last decades. China has the world’s richest reserves of REEs, sufficient to meet the global demand (18, 19). REEs are beneficial elements for plants and have been used in fertilizers in Chinese agriculture for about 40 years (20). REEs are also used in medicine. For example, rare earth-doped nanoparticles have the potential to target and treat glioblastoma (21). With the exploitation and application of REEs in multiple fields, there is an increasing possibility for humans to absorb REEs from the workplace, environment and food (22, 23). The health effects of REEs are controversial (24). While some studies found that REEs impair human health (25–33), other studies showed that higher than background concentrations of REEs in human tissues did not have significant health effects (34, 35).

Oxidative stress plays a key role in the development and progression of diabetes. The vulnerability to oxidative stress during pregnancy suggests that oxidative stress may also be an important factor in triggering GDM (35, 36). It is known that low doses of REEs have antioxidant effects, while high concentrations of REEs contribute to oxidative stress (37). This unique redox property makes rare earth oxalates show great potential for the treatment of oxidative stress-induced diseases, such as diabetes, in several animal studies (38–41). In 2011, Pourkhalili N et al. first reported the beneficial effect of the combination of cerium oxide nanoparticles/sodium selenite in diabetes treatment (42). Treatment of rats with this combination resulted in a significant reduction in blood glucose. Hence, a small intake of REEs might reduce the risk of developing GDM. However, to our knowledge, we did not find epidemiological evidence of an association between REEs exposure and GDM.

Therefore, this study aimed to investigate whether early pregnancy exposure to REEs (single metal elements or a metal mixture) is associated with the risk of GDM.

## Methods

### Study Design

This was a nested case-control study based on the Peking University Birth Cohort in Tongzhou (PKUBC-T). The primary aim of the prospective cohort was to investigate the short- and long-term health effects of pre-pregnancy and prenatal exposures on mothers and their children. This cohort has been registered in ClinicalTrials.gov (NCT 03814395, see the website for details). Baseline recruitment was conducted between June 2018 and February 2019, and a total of 5,426 pregnant women who visited the outpatient clinic for the first prenatal examination at Tongzhou maternal and child health hospital and met the following inclusion criteria were recruited: 1) age between 18 to 45 years old, 2) <14 gestational weeks, 3) resided in Tongzhou during the past half year and have no plan to move out after delivery, 4) plan to have antenatal care and delivery in Tongzhou Maternal and Child Hospital. The study was approved by the institutional review boards at Peking University (IRB00001052-18003), and all participants gave written informed consent at the enrollment.

After excluding participants who were diagnosed with diabetes before pregnancy, had a family history of diabetes, polycystic ovary syndrome, thyroid disease, hypertension, heart disease, autoimmune diseases, infectious diseases, chronic liver or kidney diseases, cigarette smoking, and alcohol consumption, a total of 200 women with GDM were selected as cases. We randomly selected 200 controls from participants without GDM. Cases and controls were matched to age ( ± 2 years old) and gestation week of taking oral glucose tolerance test (OGTT), but not to other factors.

### Data Collection

The trained nurses administrated interview questionnaires face to face to collect information including maternal characteristics and lifestyle habits at the first prenatal visit (7-13 gestational weeks), such as demographic information, pre-pregnancy weight, pregnancy history, smoking status, alcohol intake, and family history of diabetes. Dietary intake for early pregnant women was assessed using 24-hour dietary recall for two inconsecutive days within 7 days and daily intake of calories was calculated. Physical activity was evaluated using the last 7-day, short form of the International Physical Activity Questionnaire and quantified using metabolic equivalents of task (MET-min week^-1^) (43). The heights and weights of pregnant women were measured by trained nurses. Pre-pregnancy body mass index (BMI) was calculated as pre-pregnancy weight in kilograms divided by height squared in meters.

### Diagnosis of GDM

We obtained the information on GDM diagnosed by the obstetricians in the hospital from the medical records. The OGTT was implemented between 24 and 28 gestational weeks. Venous blood samples were collected at 0, 1, and 2 hours after a 75g glucose load. According to current guidelines of the American Diabetes Association (ADA) (44), women who met one or more of the following criteria were diagnosed with GDM: fasting plasma glucose ≥ 5.1mmol/L, 1 h ≥ 10.0 mmol/L, and 2 h ≥ 8.5 mmol/L.

### Metal Analysis

Blood samples were obtained from all participants at the first prenatal visit (<14 gestational weeks) in the first trimester. Samples were processed within 24 hours of collection and stored at −80°C. Fasting plasma glucose (FPG) and total vitamin D concentrations were measured using standard detection methods.

Each serum sample was transported to the laboratory on dry ice and stored at −80°C until assay. After thawing on a 4°C low-temperature console, 0.1 mL of serum sample was transferred to a 2 mL centrifuge tube. Then 0.1 mL of indium (In) internal standard and 1.8 mL of 1% nitric acid (ultrapure grade) were added, and the sample was shaken sufficiently. We measured the concentrations of elements using inductively coupled plasma mass spectrometry (ICP-MS, ELAN DRC II, PerkinElmer Sciex, USA). A total of 14 REEs were included in this study (see **Table 2**): lanthanum (La), cerium (Ce), praseodymium (Pr), neodymium (Nd), samarium (Sm), europium (Eu), gadolinium (Gd), terbium (Tb), dysprosium (Dy), holmium (Ho), erbium (Er), thulium (Tm), ytterbium (Yb) and lutetium (Lu). The limits of detection (LODs, μg/L) were 0.012 (La), 0.008 (Ce), 0.002 (Pr), 0.060 (Nd), 0.008 (Sm), 0.020 (Eu), 0.040 (Gd), 0.008 (Tb), 0.020 (Dy), 0.040 (Ho), 0.240 (Er), 0.140 (Tm), 0.020 (Yb) and 0.002 (Lu). We defined the concentration below the LOD as LOD/√2 for statistical analysis.

For quality assurance, the quantitative analysis was conducted in the Central Laboratory of Biological Elements in the Peking University Health Science Center, and the protocol was qualified by the China Metrology Accreditation (CMA) system.

### Statistical Analyses

We first performed descriptive statistical analyses of the sociodemographic, lifestyle, and clinical characteristics of the participants. Data were presented as mean ± standard deviation (SD) for normally distributed continuous variables and as median (interquartile range, IQR) for non-normally distributed continuous variables. Categorical variables were presented as frequency and percentage (N, %). The independent samples t-test and the Mann-Whitney U test were used to compare the differences between two groups for normally and non-normally distributed continuous variables, respectively. The Chi-square test was performed for the categorical variables. Because the concentrations of the elements were not normally distributed, the concentrations of all REEs were described by the median with upper and lower quartiles (P25-P75). Non-parametric analyses were used to examine the differences in REEs between the GDM group and the control group. We also conducted Spearman’s rank correlation analysis to examine correlations between different metals.

We then performed dose-response analyses. REEs concentrations were grouped based on the quartiles of their distribution among all participants (with the highest quartile as the reference). The unconditional logistic regression analysis was used to assess the association of serum REEs with the risk of GDM. The crude model only included each REEs (quartiles) without any adjustment, and the subsequent models were adjusted for pre-pregnancy BMI, education, parity, dietary energy, physical activity intensity, total vitamin D concentrations and fetal sex. K-Nearest Neighbors method was used to interpolate missing values of physical activity intensity and total vitamin D concentrations (11.8% and 5.0%, respectively). The linear trend was tested by using the median of each quartile as a continuous variable in the models.

The association of the mixture of REEs with GDM was estimated using weighted quantile sum (WQS) regression. WQS regression could combine highly correlated exposures into an index to assess the association between multiple exposures and outcomes. This method is based on the assumption that all components of the index are associated with the outcome in the same direction (all negative or all positive). After grouping different REE metals into ordinal variables (quartiles), the WQS approach was used to estimate a weighted linear index (WQS index), which identifies the significant variables in a set of multiple correlated metals and further estimates the association with GDM (45). The WQS index is a value between 0 and 1, summing to 1, determined by bootstrap sampling (n = 100). In our analysis, we set β1 as a non-constrained negative coefficient. The significance level was set at *P* < 0.05 (two-tailed test). All data analyses were conducted using R software (version 4.0.2).

## Results

### General Characteristics of Participants

The distributions of the baseline characteristics of pregnant women with GDM and controls are shown in **Table 1**. The mean age of the 400 participants was 30.24 ± 3.43 years. Compared with controls, pregnant women with GDM had a higher pre-pregnancy BMI (23.25 ± 3.62 kg/m^2^ vs. 22.50 ± 3.25 kg/m^2^, *P* = 0.029). No significant differences were observed between the cases and controls in regard to the educational level, nationality, annual household income, gravidity, parity, daily energy intake, and physical activity during early pregnancy (*P* > 0.05). For the 75g OGTT, the mean glucose levels of fasting, 1 h, and 2 h among controls were 4.68 ± 0.30, 7.20 ± 1.34, and 6.47 ± 1.03 mmol/L, respectively, while the mean levels of fasting, 1 h, and 2 h among GDM cases were 5.19 ± 0.37, 9.04 ± 1.68, and 7.69 ± 1.36 mmol/L, respectively (*P* < 0.05). Compared with controls, pregnant women with GDM had a higher total vitamin D concentrations in early pregnancy (21.40 (17.08, 26.60) ng/ml vs. 23.6 (19.18, 28.43) ng/ml, *P* = 0.020). In neonates, no differences were found between the two groups in terms of fetal sex and birth weight (*P* > 0.05).

**Table 1:** The characteristics of the study participants.

Variables	Controls (N=200)^b^	GDM Cases (N=200)^b^	*P* ^c^
**Maternal age (year), Mean±SD**	30.24±3.44	30.24±3.44	1.000
**Pre-pregnant BMI (kg/m^2^)^a^, Mean±SD**	22.50±3.25	23.25±3.62	**0.029**
**Maternal education, N (%)**			
High school or below	49 (57.6)	36 (42.4)	0.142
Collage or above	151 (47.9)	164 (52.1)	
**Nationality, N (%)**			
Han	188 (94.0)	188 (94.0)	1.000
others	12 (6.0)	12 (6.0)	
**Annual household income (Ten Thousand Yuan), N (%)**			
<10	83 (41.5)	81 (40.5)	0.560
10-20	74 (37.0)	83 (41.5)	
>20	43 (21.5)	36 (18.0)	
**Maternal gravidity, N (%)**			
1	87 (43.5)	83 (41.5)	0.762
≥2	113 (56.5)	117 (58.5)	
**Maternal parity, N (%)**			
0	114 (47.1)	128 (52.9)	0.184
≥1	86 (54.4)	72 (45.6)	
**Daily energy intake (kcal/d) in early pregnancy, median (IQR)^a^**	1348.96 (1033.65, 1670.76)	1293.98 (1027.93, 1546.80)	0.377
**Physical activity in early pregnancy, N (%)**			
Low	72 (46.5)	83 (53.5)	0.305
Middle and high	128 (52.2)	117 (47.8)	
**Total vitamin D concentrations in early pregnancy (ng/ml), median (IQR)^a^**	21.40 (17.08, 26.60)	23.6 (19.18, 28.43)	**0.020**
**Results of OGTT (mmol/L)^a^, Mean±SD**			
Fasting plasma glucose (FPG)	4.68±0.30	5.19±0.37	**<0.001**
1 h plasma glucose	7.20±1.34	9.04±1.68	**<0.001**
2 h plasma glucose	6.47±1.03	7.69±1.36	**<0.001**
**Fetal Sex, N (%)**			
Male	93 (47.2)	104 (52.8)	0.363
Female	101 (52.3)	92 (47.7)	
**Fetal weight (g), N (%)**			
<2500	5 (2.6)	10 (5.1)	0.052
2500-4000	178 (91.8)	164 (83.7)	
>4000	11 (5.7)	22 (11.2)	

^a^BMI, body mass index, OGTT, oral glucose tolerance test, IQR, Inter-quartile range.

^b^Data expressed as Mean ± SD for quantitative parametric variables, median (IQR) for non-parametric variables and N (%) for categorical variables.

^c^P-Values were calculated by independent samples t-test for normally distributed continuous variables, Mann-Whitney U test for non-normally distributed continuous variables, and Chi-square test for the category variables. Statistically significant values at P < 0.05 are shown in bold.

### The Concentrations of REEs

We compared the concentrations of REEs between two groups (see **Table 2**). La, Ce, Pr, Nd, and Sm were detected in 100% of the samples. And the detection rates of Dy and Lu were 89.25% and 80%, respectively. The least frequently detected elements were Er (0%), Tm (0%), Ho (0.25%), Yb (24.0%), Gd (42.75%), Tb (59.25%), and Eu (72.75%). Because the detection rates of these seven elements were under 80%, we excluded these metals in subsequent analyses. The median concentrations (μg/L) of the REEs in all women were 0.178 for Nd, 0.137 for Ce, 0.123 for Sm, 0.120 for Er, 0.076 for La, 0.070 for Tm, 0.040 for Dy, 0.029 for Pr, 0.027 for Eu, 0.020 for Gd, 0.020 for Ho, 0.010 for Yb, 0.009 for Tb, and 0.004 for Lu, respectively. We compared the REE concentrations in our study with those reported in other regions (see **Table S1**). No statistically significant differences in the concentrations of REEs were observed between cases and controls in this study (*P* > 0.05). The correlation coefficients between most of the REEs were significantly positive (*P* < 0.05) (see **Table S2**).

**Table 2:** Concentrations (μg/L) of the rare earth elements (REEs) in maternal blood between cases and controls.

REEs	Abbrev.	Limit of detection (LOD)	Detection Rate (%)	All (N=400)	Controls (N=200)	Cases (N=200)	*P*
				Median (IQR^a^)	Median (IQR^a^)	Median (IQR^a^)	
Lanthanum	La	0.012	100.00	0.076 (0.054-0.099)	0.075 (0.054-0.100)	0.076 (0.054-0.099)	0.986
Cerium	Ce	0.008	100.00	0.137 (0.091-0.179)	0.139 (0.103-0.174)	0.132 (0.084-0.185)	0.490
Praseodymium	Pr	0.002	100.00	0.029 (0.023-0.035)	0.030 (0.024-0.035)	0.028 (0.022-0.036)	0.334
Neodymium	Nd	0.060	100.00	0.178 (0.134-0.226)	0.179 (0.134-0.232)	0.172 (0.134-0.218)	0.342
Samarium	Sm	0.008	100.00	0.123 (0.092-0.164)	0.122 (0.091-0.167)	0.123 (0.093-0.162)	0.746
Europium	Eu	0.020	72.75	0.027 (0.010-0.036)	0.027 (0.010-0.036)	0.027 (0.010-0.035)	0.730
Gadolinium	Gd	0.040	42.75	0.020 (0.020-0.050)	0.020 (0.020-0.051)	0.020 (0.020-0.049)	0.455
Terbium	Tb	0.008	59.25	0.009 (0.004-0.012)	0.009 (0.004-0.012)	0.009 (0.004-0.012)	0.683
Dysprosium	Dy	0.020	89.25	0.040 (0.028-0.059)	0.043 (0.029-0.059)	0.039 (0.028-0.054)	0.261
Holmium	Ho	0.040	0.25	0.020 (0.020-0.020)	0.020 (0.020-0.020)	0.020 (0.020-0.020)	1.000
Erbium	Er	0.240	0.00	0.120 (0.120-0.120)	0.120 (0.120-0.120)	0.120 (0.120-0.120)	1.000
Thulium	Tm	0.140	0.00	0.070 (0.070-0.070)	0.070 (0.070-0.070)	0.070 (0.070-0.070)	1.000
Ytterbium	Yb	0.020	24.00	0.010 (0.010-0.010)	0.010 (0.010-0.021)	0.010 (0.010-0.010)	0.210
Lutetium	Lu	0.002	80.00	0.004 (0.002-0.006)	0.004 (0.002-0.006)	0.004 (0.002-0.006)	0.430

^a^IQR, Inter-quartile range.

### REEs Exposure and GDM

In order to explore the associations between REEs and GDM, we further classified the concentrations of the REEs based on quartiles (see **Table 3**). Compared with the 4th quartile, we didn’t find significant associations between REEs in other quartiles and GDM risk (*P* > 0.05). After adjusting for pre-pregnancy BMI, education, parity, dietary energy, physical activity in early pregnancy, total vitamin D concentrations and fetal sex, the associations remained not statistically significant (*P* > 0.05). Besides, no statistically significant trend was observed (*P* > 0.05).

**Table 3:** The association between the concentrations of REEs and risk of GDM.

REEs	Quartiles	GDM cases, n (%)	Controls n, (%)	UOR (95%CI)^a^	*P* value	*P* trend	AOR (95%CI)^b^	*P* value	*P* trend
La	Q4	46 (48.4)	49 (51.6)	1			1		
	Q3	55 (52.9)	49 (47.1)	1.20 (0.69-2.09)	0.529		1.29 (0.73-2.30)	0.382	
	Q2	44 (47.3)	49 (52.7)	0.96 (0.54-1.70)	0.879		1.08 (0.59-1.95)	0.810	
	Q1	49 (51.0)	47 (49.0)	1.11 (0.63-1.96)	0.717	0.841	1.22 (0.68-2.20)	0.513	0.580
Ce	Q4	57 (53.8)	49 (46.2)	1			1		
	Q3	36 (42.9)	48 (57.1)	0.65 (0.36-1.15)	0.136		0.61 (0.33-1.10)	0.102	
	Q2	39 (44.3)	49 (55.7)	0.68 (0.39-1.21)	0.190		0.75 (0.42-1.35)	0.340	
	Q1	62 (56.4)	48 (43.6)	1.11 (0.65-1.90)	0.702	0.727	1.22 (0.70-2.14)	0.482	0.462
Pr	Q4	55 (52.9)	49 (47.1)	1			1		
	Q3	35 (41.7)	49 (58.3)	0.64 (0.36-1.14)	0.127		0.58 (0.32-1.06)	0.077	
	Q2	43 (47.3)	48 (52.7)	0.80 (0.45-1.40)	0.433		0.84 (0.47-1.50)	0.550	
	Q1	61 (56.0)	48 (44.0)	1.13 (0.66-1.94)	0.652	0.722	1.21 (0.69-2.12)	0.507	0.567
Nd	Q4	38 (43.2)	50 (56.8)	1			1		
	Q3	49 (50.0)	49 (50.0)	1.32 (0.74-2.35)	0.353		1.31 (0.72-2.37)	0.375	
	Q2	57 (54.8)	47 (45.2)	1.60 (0.90-2.83)	0.109		1.57 (0.88-2.83)	0.130	
	Q1	50 (51.0)	48 (49.0)	1.37 (0.77-2.45)	0.286	0.203	1.40 (0.78-2.54)	0.263	0.191
Sm	Q4	42 (46.2)	49 (53.8)	1			1		
	Q3	56 (53.3)	49 (46.7)	1.33 (0.76-2.34)	0.316		1.27 (0.71-2.26)	0.420	
	Q2	48 (49.0)	50 (51.0)	1.12 (0.63-1.98)	0.698		1.04 (0.58-1.88)	0.898	
	Q1	48 (51.1)	46 (48.9)	1.22 (0.68-2.17)	0.504	0.585	1.23 (0.68-2.22)	0.503	0.624
Dy	Q4	40 (44.9)	49 (55.1)	1			1		
	Q3	43 (46.2)	50 (53.8)	1.05 (0.59-1.89)	0.861		0.97 (0.53-1.76)	0.912	
	Q2	57 (54.8)	47 (45.2)	1.49 (0.84-2.62)	0.173		1.41 (0.79-2.53)	0.248	
	Q1	54 (52.9)	48 (47.1)	1.38 (0.78-2.44)	0.271	0.164	1.35 (0.75-2.42)	0.317	0.200
Lu	Q4	45 (47.9)	49 (52.1)	1			1		
	Q3	40 (45.5)	48 (54.5)	0.91 (0.51-1.63)	0.744		0.89 (0.49-1.64)	0.715	
	Q2	65 (57.0)	49 (43.0)	1.44 (0.83-2.50)	0.189		1.38 (0.78-2.43)	0.268	
	Q1	44 (47.8)	48 (52.2)	1.00 (0.56-1.78)	0.995	0.555	1.10 (0.60-1.99)	0.766	0.437

^a^UOR, unadjusted odds ratio.

^b^AOR, adjusted for pre-pregnancy BMI, education, parity, dietary energy, physical activity in early pregnancy, total vitamin D concentrations and fetal sex, Cl, confidence interval.

### WQS Regression

**Figure 1:**
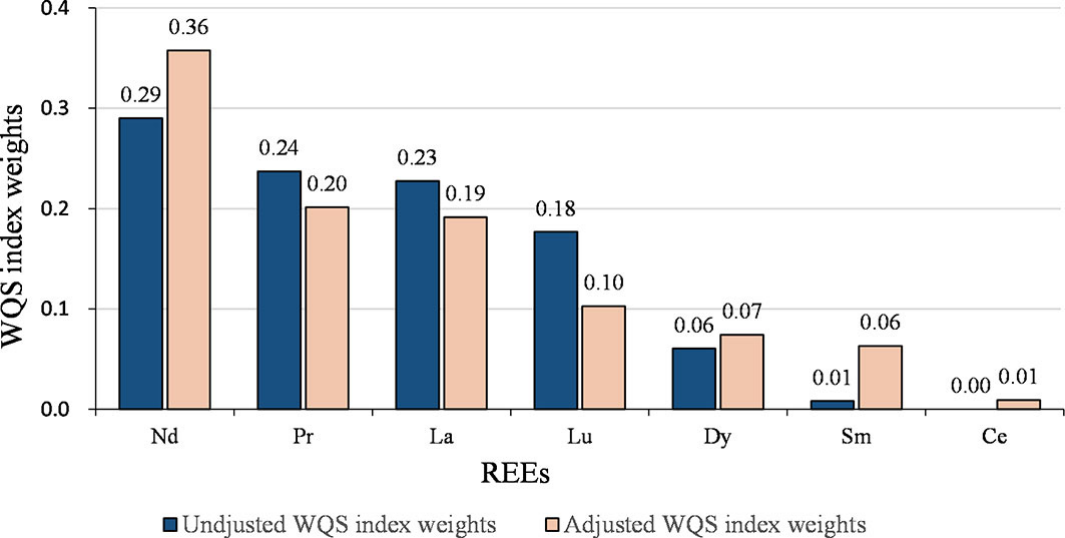
WQS weights (%) of REEs on the risk of GDM.

We calculated the WQS index for all REEs, where the contribution of each element reflected its relative effect on GDM. The detailed results are presented in **Table 4**. No statistically significant association was found in the crude model (*P* > 0.05). After adjusting for confounding variables, each quartile decrease in the WQS index was associated with a 1.67-fold (95% CI: 1.12-2.49, *P* = 0.011) higher risk of GDM. The assigned weights for each REE element are shown in **Figure 1**. Based on the weighted mean empirical weights for each REEs, the major contributors to the metal mixture index (WQS index) were Nd (35.8%), Pr (20.1%), and La (19.1%), followed by Lu (10.3%), Dy (7.4%), and Sm (6.3%), while Ce (0.9%) made almost no contribution.

**Table 4:** The association of GDM with the WQS index of REEs.

Metals^a^	WQS ORs^b^ (95% CI)	*P* value	WQS ORs^c^ (95% CI)	*P* value^d^
REEs	1.00 (0.68-1.47)	0.999	1.67 (1.12-2.49)	**0.011**
LREEs	0.99 (0.69-1.41)	0.938	1.69 (1.14-2.53)	**0.010**
HREEs	1.03 (0.80-1.32)	0.808	1.16 (0.89-1.52)	0.279

^a^REEs, all rare earth elements with detection rate >80% (including La, Ce, Pr, Nd, Sm, Dy and Lu), LREEs, light rare earth elements with detection rate >80%(including La, Ce, Pr, Nd and Sm), HREEs, heavy rare earth elements with detection rate >80% (including Dy and Lu).

^b^UOR, unadjusted odds ratio,

^c^AOR, adjusted for pre-pregnancy BMI, education, parity, dietary energy, physical activity in early pregnancy, total vitamin D concentrations and fetal sex, Cl, confidence interval.

^d^Statistically significant values at P < 0.05 are shown in bold.

**Figure 2:**
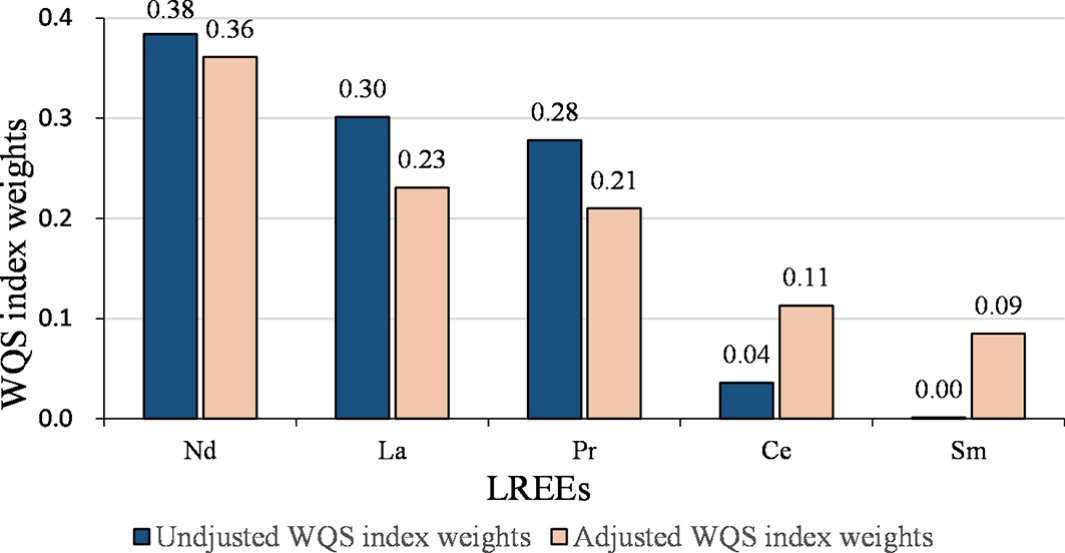
WQS weights (%) of LREEs on the risk of GDM.

The joint effect of the LREEs on GDM was similar to the results of WQS analysis for all REEs (**Table 4** and **Figure 2**). After adjusting for confounding variables, each quartile decrease in the WQS index for LREEs resulted in a 1.69-fold (95% CI: 1.14-2.53, *P* = 0.010) increase in GDM risk, with Nd (36.1%), La (23.1%), and Pr (21.0%) receiving the highest weights. However, we did not observe a significant association between WQS index for HREEs and GDM (figure not shown).

## Discussion

In this study, we investigated the association between serum REE concentrations and GDM risk among pregnant women in Beijing, China. We didn’t observe a significant association between REEs and GDM by logistic regression analysis. However, the WQS model indicated that exposure to lower levels of REEs mixtures was associated with an increased risk of GDM, and three elements, Nd, Pr, and La, were important contributors to the REE mixture.

Blood concentrations of REEs in unexposed populations vary considerably by race, region, age, gender, and the different analytical methods used (28). As shown in **Table S1**, previous studies (28, 31, 46–49) showed that the range of the median values was 0.01-0.854 μg/L for La, 0.02-2.546 μg/L for Ce, 0.01-0.132 μg/L for Pr, 0.01-0.839 μg/L for Nd, 0.01-0.132 μg/L for Sm, 0.01-0.034 μg/L for Eu, 0.049-0.059 μg/L for Gd, 0.001-0.011 μg/L for Tb, 0.01-0.047 μg/L for Dy, 0.001-0.01 μg/L for Lu, 0.01-0.529 μg/L for Yb among the non-occupationally exposed population in China, Spain, Romania, and Sub-Saharan Africa. The concentrations of all the serum REEs in our study fell in these ranges. The high detection rates in blood for most REEs in humans are remarkable, considering that to date there are very few references in the scientific literature dealing with the effects of chronic exposure to REEs on human health (24–28). Therefore, it remains unclear whether the exposure to REEs during pregnancy is related to GDM or not.

In the present study, we didn’t observe a significant association between individual REEs and GDM risk. When we classified the concentrations of the REEs according to quartiles, we found no statistically significant association. Considering the absence of clear toxicological criteria, some authors analyzed the combined effects of these elements, as they were often used in combination in the manufacture of multiple devices (50). The WQS model can not only examine the effects of the REE mixture but also identify those elements most strongly associated with GDM (45). In WQS analysis, lower levels of REE mixture were significantly associated with a higher risk of GDM, and Nd, Pr and La were likely to be the most important contributors. The joint effect of the LREEs on GDM was similar to the results of WQS analysis for all REEs. A possible explanation for the similar results is that the three most important contributors (Nd, Pr, and La) belong to LREEs. To the best of our knowledge, there is no published report in the literature on REEs in maternal serum and risk for GDM. A previous study conducted in Shanxi province of China reported that higher concentrations of REEs were associated with an increased risk of hypertension (24). Two studies conducted in Shanxi and Hebei provinces of China found that high concentrations of REEs were associated with increased risks of fetal neural tube defects (NTDs) (25, 26). Besides, Liu et al. found that increased maternal urinary REEs (Ce and Yb) were associated with decreased neonatal thyroid-stimulating hormone (TSH) levels (27). Another study suggested that REEs might play a role in the development of anemia (28). Liu et al. showed that prenatal exposure to REEs and REE mixtures were associated with an increased risk of premature rupture of membranes (29). Liu et al. found that *in utero* exposure to REE mixtures increased the risk of orofacial clefts (30). According to these studies, exposure to higher levels of REEs was a risk factor for some diseases, while in our study, exposure to higher levels of REEs might be a protective factor for GDM.

The possible underlying mechanisms of this inconsistency are as follows. REEs may act either as antioxidants or pro-oxidants, depending on the environment, the nature of the bonding in their compounds, and their concentration in the tissues (51). Results of previous studies might be explained by oxidative stress induced by exposure to REEs. However, some REEs have been reported to have antioxidant effects in clinical applications (52). For example, cerium oxide nanoparticles could exhibit superoxide dismutase (SOD) activity and act as a free radical scavenger (42). GDM is usually accompanied by increased levels of oxidative stress and inadequate antioxidant defense responses (36). Therefore, REEs may reduce oxidative stress associated with GDM. Moreover, individual REEs and REE mixtures have been shown to have hormetic effects on several health endpoints. In other words, REEs can increase or improve biological events (e.g. growth) at low concentrations and exhibit inhibitory or toxic effects at increasing doses/concentrations (35). Another possible explanation is related to the relatively low concentrations of REEs in our study. Low concentrations of REEs might stimulate pancreatic beta (β) cells to secrete insulin to regulate glucose metabolism and maintain blood glucose stability (53–55). Within a certain range of concentrations, exposure to lower concentrations of REEs might be a risk factor for GDM. Further studies are needed to validate the role of REEs in the pathophysiology of GDM.

To the best of our knowledge, this study is the first to evaluate the association between early pregnancy exposure to REEs and the risk of GDM. This is one of the strengths of our study. Second, comprehensive information regarding potential confounders was prospectively collected in the birth cohort and controlled in our statistical analyses. Third, the sample size of this prospective cohort study was relatively large, and we collected blood samples in early pregnancy to evaluate metal exposure, which meets the criterion of temporality for causal inference. Finally, the information in our questionnaire was collected using standardized interview procedures by trained local health workers at the hospital. However, there were some limitations in our study. First, causality cannot be made based on the findings of the current study because of the observational nature of the study design. Second, everyone is exposed to multiple toxic substances in real life, and it is difficult to fully rule out the effects of residual confounders. Thirdly, the data of dietary intake were retrospectively collected and had recall bias, causing underestimation of energy intake in this study. However, we think that the underestimation of energy intake may not affect the main results, because it was a covariate in our study. More validation studies are needed in the future.

## Conclusion

In conclusion, we observed that early pregnancy exposure to lower levels of REE mixtures increased the risk of GDM. Nd, Pr, and La showed the greatest contribution to the metal mixture index related to GDM. Our findings suggested that REEs are more likely to play a role through oxidative stress or other pathways. Animal studies are needed to uncover the possible mechanisms underlying the association between the mixture of REEs and GDM risk. The results of this study warrant further epidemiological investigation in other populations.

## Data Availability Statement

The original contributions presented in the study are included in the article/**Supplementary Material**. Further inquiries can be directed to the corresponding authors.

## Ethics Statement

The studies involving human participants were reviewed and approved by the institutional review boards at Peking University (IRB00001052-18003). The patients/participants provided their written informed consent to participate in this study.

## Author Contributions

Conceptualization, XX, YW, LY, XM, and H-JW. Methodology, XX, YW, SL, ZL, and HB. Software, XX and XY. Validation, XY and H-JW. Formal Analysis, XX, YW, and YJ. Data Curation, NH, XY, JL, CJ, LL, and SZ. Writing - Original Draft Preparation, XX and YW. Writing–Review and Editing, LY, HW, and BW. Supervision, LY, XM, and H-JW. Project Administration, XX. Funding Acquisition, H-JW. All authors have read and agreed to the published version of the manuscript.

## Funding

This study was funded by the National Key Research and Development Program (No. 2016YFC1000300 and No. 2016YFC1000307) and National Natural Science Foundation of China (81973053 and 81703240).

## Conflict of Interest

The authors declare that the research was conducted in the absence of any commercial or financial relationships that could be construed as a potential conflict of interest.

## Publisher’s Note

All claims expressed in this article are solely those of the authors and do not necessarily represent those of their affiliated organizations, or those of the publisher, the editors and the reviewers. Any product that may be evaluated in this article, or claim that may be made by its manufacturer, is not guaranteed or endorsed by the publisher.
