# Supplementary material for: Early Pregnancy Exposure to Rare Earth Elements and Risk of Gestational Diabetes Mellitus: A Nested Case-Control Study
Source: Front Endocrinol (Lausanne). 2021 Dec 20;12:774142. doi: 10.3389/fendo.2021.774142 (PMC8721846; doi:10.3389/fendo.2021.774142)
Supplement: Supplementary file 1 [file DataSheet_1.pdf]

Supporting Information

Table S1 Comparison of concentrations (μg/L) of the ten rare earth elements in serum or blood with the previous studies in China and other countries or regions

References	This study[a]		Guo et al. 2020[a]		Wei et al. 2020[a]		Bao et al. 201[a]		Bai et al. 2019[b]		Badea et al. 2018[c]			Henriquez-Hernandez et al. 2017[a]		Cabrera-Rodríguez et al.2018[a]
Sampling year	2018		2017		2010–2018		2017		/		2017.12–2018.2			2010		2015.3-2016.4
Country or region	Beijing, China		Taizhou, Jiangsu Province, China		Shanxi Province, China		Baotou, China		Henan, China		Romania			Sub-Saharan(immigrants)		La Palma (Canary Islands, Spain).
Sample Type/Site	serum		Blood		serum		blood		blood		serum			blood		umbilical cord blood
	GDM case	Control	NTD case	Control	NTD case	Control	Nearer to the mining area	Control area	Exposure	Control	Cigarette smokers	E-cigarette users	Non-smokers	Anemic group	Non-anemic group	Whole series
Gender	Female	Female	Male/Female	Male/Female	Female	Female	/	/	/	/	/	/	/	/	/	/
Pollution source	/	/	/	/	/	/	mine	/	chromate	/	Cigarette	E-cigarette	/	/	/	/
La	0.076 (0.054-0.099)	0.075 (0.054-0.100)	0.479 (0.078-0.679)	0.032 (0.026-0.042)	0.072 (0.052-0.107)	0.059 (0.045-0.083)	0.854 (0.702-1.061)	0.700 (0.554-0.827)	0.078 (0.106)	0.051 (0.050)	0.14 (0.10-0.32)	0.12 (0.11-0.22)	/	0.01 (0.01-0.02)	/	0.01（0.01-0.03）
Ce	0.132 (0.084-0.185)	0.139 (0.103-0.174)	2.546 (1.789-3.538)	0.017 (0.012-0.026)	0.116 (0.080-0.233)	0.090 (0.063-0.130)	1.724 (1.446-2.498)	1.474 (0.952-1.807)	0.205 (0.2082)	0.123 (0.169)	0.14 (0.10-0.76)	0.21 (0.17-0.31)	/	0.02 (0.02-0.03)	/	0.03（0.02-0.06）
Pr	0.028 (0.022-0.036)	0.030 (0.024-0.035)	0.020 (0.014-0.026)	0.011 (0.008-0.017)	0.030 (0.023-0.043)	0.028 (0.022-0.037)	0.132 (0.110-1.165)	0.097 (0.082-1.140)	0.015 (0.026)	0.010 (0.006)	0.01 (0.00-0.05)	0.02 (0.01-0.05)	0.01 (0.00-0.06)	0.01 (0.01-0.02)	/	/

Nd	0.172 (0.134-0.218)	0.179 (0.134-0.232)	0.085 (0.072-0.106)	0.082 (0.067-0.125)	0.212 (0.156-0.265)	0.195 (0.150-0.252)	0.839 (0.587-1.201)	0.621 (0.457-0.815)	0.024 (0.050)	0.016 (0.021)	0.03 (0.02-0.30)	0.05 (0.02-0.13)	0.02 (0.02-0.06)	0.05 (0.04-0.06)	0.01 (0.01-0.02)	0.01（<LOQ-0.01）
Sm	0.123 (0.093-0.162)	0.122 (0.091-0.167)	0.049 (0.039-0.063)	0.056 (0.046-0.074)	0.132 (0.098-0.173)	0.127 (0.096-0.168)	/	/	0.009 (0.013)	0.004 (0.006)	0.01 (0.01-0.05)	0.02 (0.01-0.03)	0.01 (0.01-0.04)	0.02 (0.01-0.03)	0.01 (0.01-0.02)	/
Eu	0.027 (0.010-0.035)	0.027 (0.010-0.036)	0.027 (0.023-0.031)	0.026 (0.021-0.029)	0.034 (0.023-0.048)	0.030 (0.022-0.042)	/	/	0.007 (0.009)	0.005 (0.002)	0.00 (0.00-0.02)	0.00 (0.00-0.01)	0.01 (0.00-0.05)	0.02 (0.01-0.03)	/	0.01（<LOQ-0.02）
Gd	0.020 (0.020-0.049)	0.020 (0.020-0.051)	0.059 (0.048-0.073)	0.049 (0.046-0.115)	/	/	/	/	/	/	/	/	/	/	/	/
Tb	0.009 (0.004-0.012)	0.009 (0.004-0.012)	/	/	0.011 (0.004-0.014)	0.010 (0.004-0.013)	/	/	0.002 (0.002)	0.001 (0.002)	/	/	0.01 (0.01-0.05)	/	/	/
Dy	0.039 (0.028-0.054)	0.043 (0.029-0.059)	0.026 (0.022-0.032)	0.029 (0.022-0.037)	0.047 (0.031-0.063)	0.043 (0.030-0.061)	/	/	0.017 (0.021)	0.013 (0.007)	0.01 (0.01-0.02)	0.01 (0.01-0.02)	0.01 (0.01-0.05)	/	/	/
Ho	0.020 (0.020-0.020)	0.020 (0.020-0.020)	/	/	/	/	/	/	/	/	/	/	/	/	/	/
Er	0.120 (0.120-0.120)	0.120 (0.120-0.120)	/	/	/	/	/	/	/	/	/	/	/	/	/	/
Tm	0.070 (0.070-0.070)	0.070 (0.070-0.070)	/	/	/	/	/	/	/	/	/	/	/	/	/	/
Yb	0.010 (0.010-0.010)	0.010 (0.010-0.021)	/	/	/	/	/	/	/	/	/	/	/	/	/	/
Lu	0.004 (0.002-0.006)	0.004 (0.002-0.006)	0.003 (0.002-0.003)	0.003 (0.002-0.004)	0.006 (0.004-0.008)	0.005 (0.003-0.007)	/	/	0.001 (0.0018)	0.001 (0.001)	0.01 (0.01-0.01)	0.00 (0.00-0.00)	0.01 (0.00-0.05)	/	/	/

Yb	/	/	/	/	0.529 (0.377-0.794)	0.458 (0.339-0.738)	/	/	0.542 (0.661)	0.439 (0.143)	0.03 (0.02-0.21)	0.04 (0.01-0.08)	0.02 (0.02-0.04)	/	/	0.01（<LOQ-0.01）

*a Metal concentrations were shown as median (25th - 75th percentile);
b Metal concentrations were shown as median (IQR, interquartile range);
c Metal concentrations were shown as median (5th - 95th percentile).

Table S2 Correlations between REEs among the participants (n=400)

r	La	Ce	Pr	Nd	Sm	Eu	Gd	Tb	Dy	Ho	Er	Tm	Yb	Lu
**La**	1.000													
**Ce**	0.615*	1.000												
**Pr**	0.324*	0.323*	1.000											
**Nd**	0.302*	0.303*	0.540*	1.000										
**Sm**	0.016	0.028	0.373*	0.536*	1.000									
**Eu**	0.038	0.010	0.354*	0.456*	0.557*	1.000								
**Gd**	0.220*	0.232*	0.421*	0.499*	0.378*	0.389*	1.000							
**Tb**	0.031	0.027	0.296*	0.470*	0.549*	0.487*	0.373*	1.000						
**Dy**	-0.038	-0.064	0.332*	0.430*	0.551*	0.470*	0.337*	0.489*	1.000					
**Ho**	-0.028	-0.033	0.048	0.065	0.026	0.020	-0.035	0.044	0.098	1.000				
**Er**	—	—	—	—	—	—	—	—	—	—	1.000			
**Tm**	—	—	—	—	—	—	—	—	—	—	—	1.000		
**Yb**	0.069	0.091	0.259*	0.377*	0.327*	0.292*	0.308*	0.332*	0.329*	-0.025	—	—	1.000	
**Lu**	-0.032	-0.007	0.317*	0.438*	0.501*	0.446*	0.323*	0.410*	0.396*	0.078	—	—	0.317*	1.000

$^{*}p$-Value < 0.001

Pearson correlation coefficients were shown in the table
